# The genetic and environmental hierarchical structure of anxiety and depression in the UK Biobank

**DOI:** 10.1002/da.22991

**Published:** 2020-01-17

**Authors:** Genevieve Morneau‐Vaillancourt, Jonathan R. I. Coleman, Kirstin L. Purves, Rosa Cheesman, Christopher Rayner, Gerome Breen, Thalia C. Eley

**Affiliations:** ^1^ Research Unit on Child Psychosocial Maladjustment, École de Psychologie, Faculté des sciences sociales Université Laval Quebec City Quebec Canada; ^2^ Social, Genetic, and Developmental Psychiatry Centre, Institute of Psychiatry, Psychology and Neuroscience King's College London London UK; ^3^ National Institute of Health Research Biomedical Research Centre South London and Maudsley NHS Trust London UK

**Keywords:** anxiety, depression, genetics, loneliness, social isolation

## Abstract

**Background:**

Anxiety and depressive disorders can be classified under a bidimensional model, where depression and generalized anxiety disorder are represented by distress and the other anxiety disorders, by fear. The phenotypic structure of this model has been validated, but twin studies only show partial evidence for genetic and environmental distinctions between distress and fear. Moreover, the effects of genetic variants are mostly shared between anxiety and depression, but the genome‐wide genetic distinction between distress and fear remains unexplored. This study aimed to examine the degree of common genetic variation overlap between distress and fear, and their associations with the psychosocial risk factors of loneliness and social isolation.

**Methods:**

We used genome‐wide data from 157,366 individuals in the UK Biobank who answered a mental health questionnaire.

**Results:**

Genetic correlations indicated that depression and generalized anxiety had a substantial genetic overlap, and that they were genetically partially distinct from fear disorders. Associations with loneliness, but not social isolation, showed that loneliness was more strongly associated with both distress disorders than with fear.

**Conclusions:**

Our findings shed light on genetic and environmental mechanisms that are common and unique to distress and fear and contribute to current knowledge on individuals’ susceptibility to anxiety and depression.

## 1 INTRODUCTION

Anxiety and depressive disorders are the leading mental health problems contributing to the world disability burden (Kessler et al., 2009). Both groups of disorders have heterogeneous symptoms and frequently co‐occur, calling their distinction into question (APA, 2013). One proposed solution to this question is a bidimensional hierarchical structure of distress and fear (Krueger, 1999; Watson, 2005). Preliminary evidence for this model comes from the findings that depression is more often comorbid with generalized anxiety disorder than with other anxiety disorders, and that depression and generalized anxiety share a core component of negative affect symptoms (Mineka, Watson, & Clark, 1998). As a result, depression and generalized anxiety have been characterized as distress disorders. The other anxiety disorders, social anxiety, agoraphobia, specific phobia, and panic disorder have consequently been classified under a fear dimension. The phenotypic structure of this model has been validated at both the symptom and disorder levels (Slade & Watson, 2006; Vollebergh et al., 2001). However, to fully explore this proposed structure, it is necessary to understand the etiological mechanisms underlying both dimensions of distress and fear. One approach to validating this model is to examine the extent to which genetic and environmental factors are shared both within and across these dimensions.

### 1.1 Genetic structure of distress and fear

Anxiety and depressive disorders are influenced by genetic factors (Craske et al., 2017; Sullivan, Neale, & Kendler, 2000). Previous twin studies showed partial evidence for a genetic structure supporting the distress and fear model (Kendler, Prescott, Myers, & Neale, 2003; Waszczuk, Zavos, Gregory, & Eley, 2014). This support mainly comes from two findings. The first is that generalized anxiety and depression have a substantial genetic overlap, suggesting that distress disorders have a common genetic vulnerability (Kendler, Neale, Kessler, Heath, & Eaves, 1992; Roy, Neale, Pedersen, Mathé, & Kendler, 1995). The second is that disorders across the distress and fear dimensions are partly genetically distinguishable (Kendler et al., 2003; Waszczuk et al., 2014). Therefore, each dimension has specific genetic influences. However, genetic findings from twin studies do not entirely support the distress and fear model. Notably, fear disorders such as panic disorder and agoraphobia share some genetic risk with distress disorders (Hettema, Prescott, Myers, Neale, & Kendler, 2005; Kendler et al., 2003). Also, the genetic links among fear disorders remain unclear, with some studies indicating common genetic influences and others suggesting disorder‐specific genetic effects (Eley, Rijsdijk, Perrin, O'Connor, & Bolton, 2008; Loken, Hettema, Aggen, & Kendler, 2014; Silberg, Rutter, & Eaves, 2001; Waszczuk et al., 2014). In sum, twin studies only partially support the distress and fear model from a genetic perspective.

One limit of twin studies is that they do not identify the specific genetic variants involved. Genome‐wide association studies (GWAS) explore common genetic variation across the whole genome, enabling identification of associations between specific genetic variants and disorders. Studies using this approach are beginning to show that common genetic variants (single‐nucleotide polymorphism; SNP) account for individual differences in depression and anxiety. Findings indicate that SNP‐based heritability of anxiety and depression varies between 0.10 and 0.30 (Howard et al., 2019; Meier et al., 2019; Otowa et al., 2016; Purves et al., 2019; Wray et al., 2018). SNP heritability is one way to show the cumulative contribution of a subset of SNPs to a trait's total heritability (Bulik‐Sullivan, Loh et al., 2015). These recent GWAS also showed that these SNPs were located at various regions on the genome. Therefore, the cumulative effect of these SNPs partially explains individual differences in anxiety and depression. Of more relevance to the current question are findings of genome‐wide genetic correlations showing considerable overlap in genome‐wide SNPs linked to anxiety and depression. Correlation estimates range between 0.60 and 0.80 (Anttila et al., 2018; Meier et al., 2019; Purves et al., 2019). However, these genome‐wide genetic correlations were based on general measures of anxiety and to date, such correlation between generalized anxiety and the fear disorders remain unexplored. Therefore, no study has yet examined common genetic variation with regard to understanding the relationship between distress and fear dimensions.

### 1.2 Environmental structure of distress and fear

In addition to genetic influences, twin studies have also identified substantial environmental contributions to anxiety and depressive disorders (Eley et al., 2008; Kendler et al., 2003; Loken et al., 2014; Roy et al., 1995; Silberg et al., 2001; Waszczuk et al., 2014). However, in line with genetic findings from twin studies, their findings are equivocal with respect to defining the underlying etiology of the distress and fear model. Furthermore, twin studies did not allow identifying specific aspects of the environment that may contribute to anxiety and depression.

One way to further explore the role of the environment in the structure of distress and fear is to investigate associations with specific types of psychosocial stress. Two factors reflecting psychosocial stress that have recently been associated with several health issues, including anxiety and depression, are loneliness and social isolation (Baselmans et al., 2019; Beutel et al., 2017; Holt‐Lunstad, Smith, Baker, Harris, & Stephenson, 2015; Hyland et al., 2018; Luo, Hawkley, Waite, & Cacioppo, 2012). Loneliness is the subjective feeling of a dissatisfaction with actual and desired social interactions (Ernst & Cacioppo, 1999), and social isolation is the objective lack of social interactions (Coyle & Dugan, 2012). With respect to their role in clarifying the distress and fear model, loneliness may be common to distress disorders since it is associated with both depression and generalized anxiety (Beutel et al., 2017; Hyland et al., 2018). However, loneliness has also been linked to panic attacks (Beutel et al., 2017). Therefore, its role in distinguishing distress from fear is unclear. Furthermore, there is currently conflicting evidence as to whether social isolation is associated with anxiety and depression. Some studies suggest that it is associated with social anxiety and depression (Schwarzbach, Luppa, Forstmeier, König, & Riedel‐Heller, 2014; Teo, Lerrigo, & Rogers, 2013), whereas others found no such association (Coyle & Dugan, 2012; Steptoe, Shankar, Demakakos, & Wardle, 2013). At present, we do not know whether loneliness and social isolation have independent relationships with distress and fear disorders since no study has yet examined and compared their respective associations with all anxiety and depressive disorders.

### 1.3 Objectives of the study

The aim of this study was to verify the distress and fear model by investigating specific genetic and environmental factors on depression and generalized anxiety disorder (distress), and all other anxiety disorders (fear). The first objective was to examine genome‐wide genetic overlap among distress disorders, and then to see whether this genetic risk was distinct from that on fear disorders. The second objective was to investigate the extent to which associations with loneliness and social isolation supported this hierarchical structure.

## 2 MATERIALS AND METHODS

### 2.1 Participants

Participants were from the UK Biobank, a large nation‐wide study documenting general health and illness of more than 500,000 adults aged 40–69 years at recruitment (see Sudlow et al., 2015 for more details on sample and recruitment). This study included a subset of 157,366 participants who completed a mental health follow‐up questionnaire approximately 6–8 years after recruitment (see Davis et al., 2018 for a description of this questionnaire).

### 2.2 Measurements

#### 2.2.1 Anxiety and depressive disorders

Participants were separated into cases and controls for all disorders.

Disorder diagnoses for depression and generalized anxiety were derived from the definition from Davis et al. (2018). Cases met probable lifetime criteria based on questions from the Composite International Diagnostic Interview short‐form (Kessler, Andrews, Mroczek, Ustun, & Wittchen, 1998). Controls did not meet these criteria and were excluded if they reported taking antidepressant medication.

To assess all other anxiety disorders, participants answered the following question: *Have you been diagnosed with one or more of the following mental health problems by a professional, even if you don't have it currently?* Fear cases were participants who had been diagnosed with at least one of these disorders: social anxiety, agoraphobia, specific phobia, or panic. This combination allowed us (a) to distinguish the fear disorders from generalized anxiety and (b) to have sufficient statistical power to perform genomic analyses. The number of cases for each fear disorder was not large enough to examine each one separately. When participants did not report any diagnosis and did not report taking antidepressant medication, they were considered controls.

#### 2.2.2 Loneliness and social isolation

Participants were considered lonely if they answered “Yes” to the following question: *Do you often feel lonely?* They were socially isolated if they were living alone, did not attend a social activity once a week or more often, and/or had friends or family visit them once every few months or less. Both scales were comparable to previously used validated evaluations (e.g., Hughes, Waite, Hawkley, & Cacioppo, 2004; Tanskanen & Anttila, 2016). More details on these measures are provided in the Supplementary material.

#### 2.2.3 Genetic data

Genome‐wide genotyping and imputation for the UK Biobank are described in Bycroft et al. (2018). Quality control procedures were applied to the genetic data and are described in Coleman et al. (2019). SNPs that were genotyped or imputed from the reference panel of the Haplotype Reference Consortium with an INFO score greater than 0.4, and which had a minor allele frequency greater than 0.01 were included in the analyses. Individuals with gender discordant genetic information, unusual missingness, or heterozygosity, or who were up to third‐degree relatives and closer were not included. The final sample included individuals of White Western European ancestry to limit population structure confounding.

### 2.3 Analytical approach

We first verified the phenotypic structure of the distress and fear model by conducting two sets of correlational analyses in R 3.3.3 (R Core Team, 2017). First, we conducted tetrachoric correlations between depression, generalized anxiety, and fear. Then, disorders were regressed on each other, one at a time, to isolate unique parts of variance (see Table 1 for a description of all phenotypes). We conducted partial correlations using residual phenotypes to examine unique associations between pairs of disorders.

**Table 1:** Phenotype variables

Initial phenotypes	Residual phenotypes^a^		
Depression	Depression‐(GAD)	Depression‐(fear)	Depression‐(GAD + fear)
GAD	GAD‐(depression)	GAD‐(fear)	GAD‐(Depression + fear)
Fear	Fear‐(GAD)	Fear‐(depression)	Fear‐(GAD + depression)

Abbreviation: GAD, generalized anxiety disorder.

^a^ Outcome‐(covariate[s]).

Examining the genetic structure of distress and fear requires information about the individual effects of each SNP on a trait, and these effects are obtained by conducting a GWAS. Therefore, preliminary to main analyses, we conducted GWAS with BGENIE version 1.2 for all disorder phenotypes presented in Table 1, controlling for age, sex, six genetic principal components for European samples, and factors representing variability in initial assessment center and genotyping batch (Bycroft et al., 2017). GWAS results were not interpreted and were used for the following genetic correlation analyses (summary statistics of GWAS are available at https://phenviz.navigome.com/downloads). With GWAS results, we estimated (a) SNP heritability to document the contribution of common genetic variation to each disorder and (b) genetic correlations between disorders (Bulik‐Sullivan, Finucane et al., 2015; Bulik‐Sullivan, Loh et al., 2015). We conducted genetic correlations with the initial and residual phenotypes that were used for full and partial phenotypic correlations. We also conducted genetic correlations between loneliness, social isolation, and disorders (see Figures S1 and S2).

Finally, to investigate the environmental structure of distress and fear disorders, we examined their full and partial phenotypic correlations with loneliness and social isolation. Here, partial correlations allowed examining the extent to which controlling for one disorder affected the association between another disorder and one psychosocial factor.

## 3 RESULTS

### 3.1 Phenotype description

The number of cases and controls for each phenotype is provided in Table 2. Loneliness and social isolation were evaluated in the full sample of the UK Biobank, unlike anxiety and depressive disorders that were measured with the Mental Health Questionnaire on a subset of the sample. This explains why there were more participants for loneliness and social isolation.

**Table 2:** Distributions of disorders

	Depression	Generalized anxiety	Fear	Loneliness	Social isolation
Cases	44,357	11,111	11,425	88,430	15,530
Controls	88,650	96,821	114,853	389,807	487,409
Cases/cases + controls	0.33	0.10	0.09	0.18	0.03
Total number of subjects	133,007	107,932	126,278	478,237	502,939

### 3.2 Phenotypic structure of distress and fear

Full and partial correlations were used to examine phenotypic associations among disorders (see Table 3). Full correlations partially supported the distress and fear structure. However, the phenotypic structure of distress and fear emerged more clearly when reviewing partial correlations. Differences between correlations were tested with a two‐sample Z test after performing a Fisher r‐to‐z transformation. All partial correlations were statistically different from each other, and from their corresponding full correlation. These associations provide preliminary evidence for the phenotypic distinction between distress and fear dimensions.

**Table 3:** Phenotypic correlations between disorders

	Generalized anxiety	Depression	Fear
Generalized anxiety	–	0.872 (0.868–0.878)	0.723 (0.715–0.733)
Depression	0.561 (0.557–0.566)	–	0.510 (0.500–0.522)
Fear	0.268 (0.262–0.274)	0.056 (0.050–0.063)	–

*Note*: Full correlations are presented in the right part of the table above the diagonal. Partial correlations, which controlled for the other phenotype, are bottom left of the diagonal. All correlations are statistically significant, *p* < .001. 95% confidence intervals are presented in parentheses and were bootstrapped for tetrachoric correlations.

### 3.3 Genetic structure of distress and fear

Table 4 presents SNP heritability estimates for all variables. Liability scale estimates were based on sample prevalence under the assumption of accurate sampling. Sensitivity analyses were conducted, with population prevalence 10% higher or lower than sample prevalence. Heritability for psychosocial factors were included because we tested their genetic correlations with the disorders, and these correlations are best understood in the context of their individual SNP heritability (see the Supplementary material).

**Table 4:** SNP heritability estimates

Phenotype	Observed *h* ^2^	*h* ^2^ converted to liability scale		
		Population prevalence = sample prevalence	Population prevalence = sample prevalence + 10%	Population prevalence = sample Prevalence − 10%
Generalized anxiety	0.058 (0.006)	0.215 (0.022)	0.224 (0.023)	0.205 (0.021)
Depression	0.070 (0.006)	0.124 (0.010)	0.128 (0.010)	0.121 (0.010)
Fear	0.028 (0.004)	0.103 (0.015)	0.107 (0.015)	0.098 (0.014)
Loneliness	0.036 (0.002)	0.078 (0.004)	0.081 (0.004)	0.075 (0.004)
Social isolation	0.007 (0.004)	0.042 (0.025)	0.045 (0.026)	0.043 (0.025)

*Note*: Standard errors are presented in parentheses. All heritability estimates are statistically significant, *p* < .001.

Abbreviation: SNP, single‐nucleotide polymorphism.

Genetic correlations are presented in Table 5 and indicated that all disorders were all genetically correlated. Partial correlations showed a pattern of associations that, again, partially supported the distress and fear distinction. Depression and generalized anxiety remained strongly genetically correlated after controlling for fear, and their respective genetic association with fear was reduced after controlling for generalized anxiety (for depression) or depression (for generalized anxiety). Yet, despite being slightly attenuated, the genetic correlation between generalized anxiety and fear remained strong after controlling for depression, therefore not entirely distinguishing fear from distress disorders. Finally, analyses using the block‐jackknife method, a resampling technique used to estimate standard errors (Bulik‐Sullivan, Finucane et al., 2015; Quenouille, 1956; Tukey, 1958) showed that none of these genetic correlations were statistically different.

**Table 5:** Genetic correlations between disorders

	Generalized anxiety	Depression	Fear
Generalized anxiety	–	0.816 (0.050)	0.718 (0.076)
Depression	0.798 (0.059)	–	0.679 (0.069)
Fear	0.632 (0.191)	0.335 (0.264)	–

*Note*: Full correlations are presented in the right part of the table above the diagonal. Partial correlations, which controlled for the other phenotype, are bottom left of the diagonal. All correlations are statistically significant, *p* < .001. Standard errors are presented in parentheses.

### 3.4 Environmental structure of distress and fear

**Figure 1:**
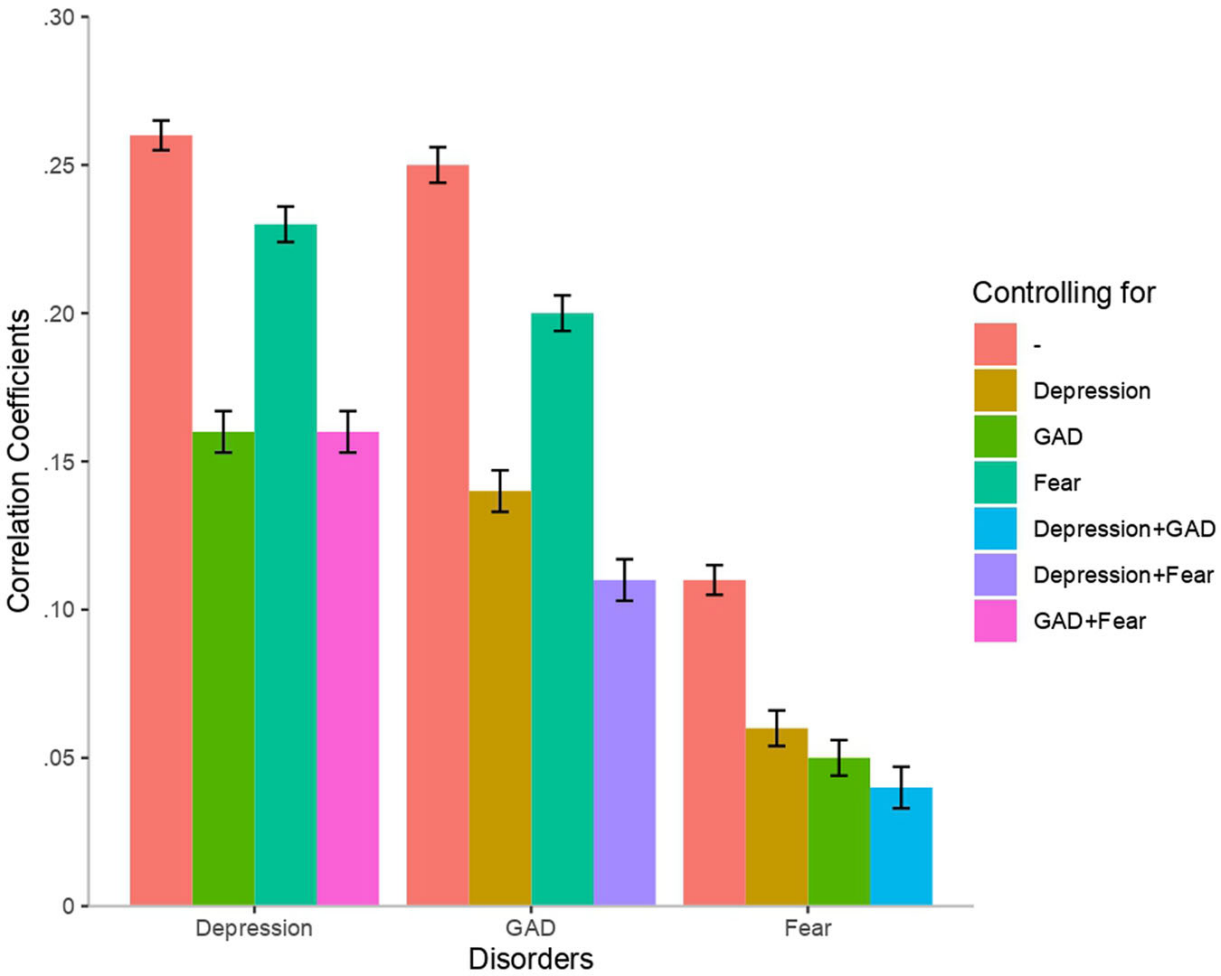
Phenotypic correlations between loneliness and disorders. 95% confidence intervals are presented as error bars. GAD, generalized anxiety disorder

**Figure 2:**
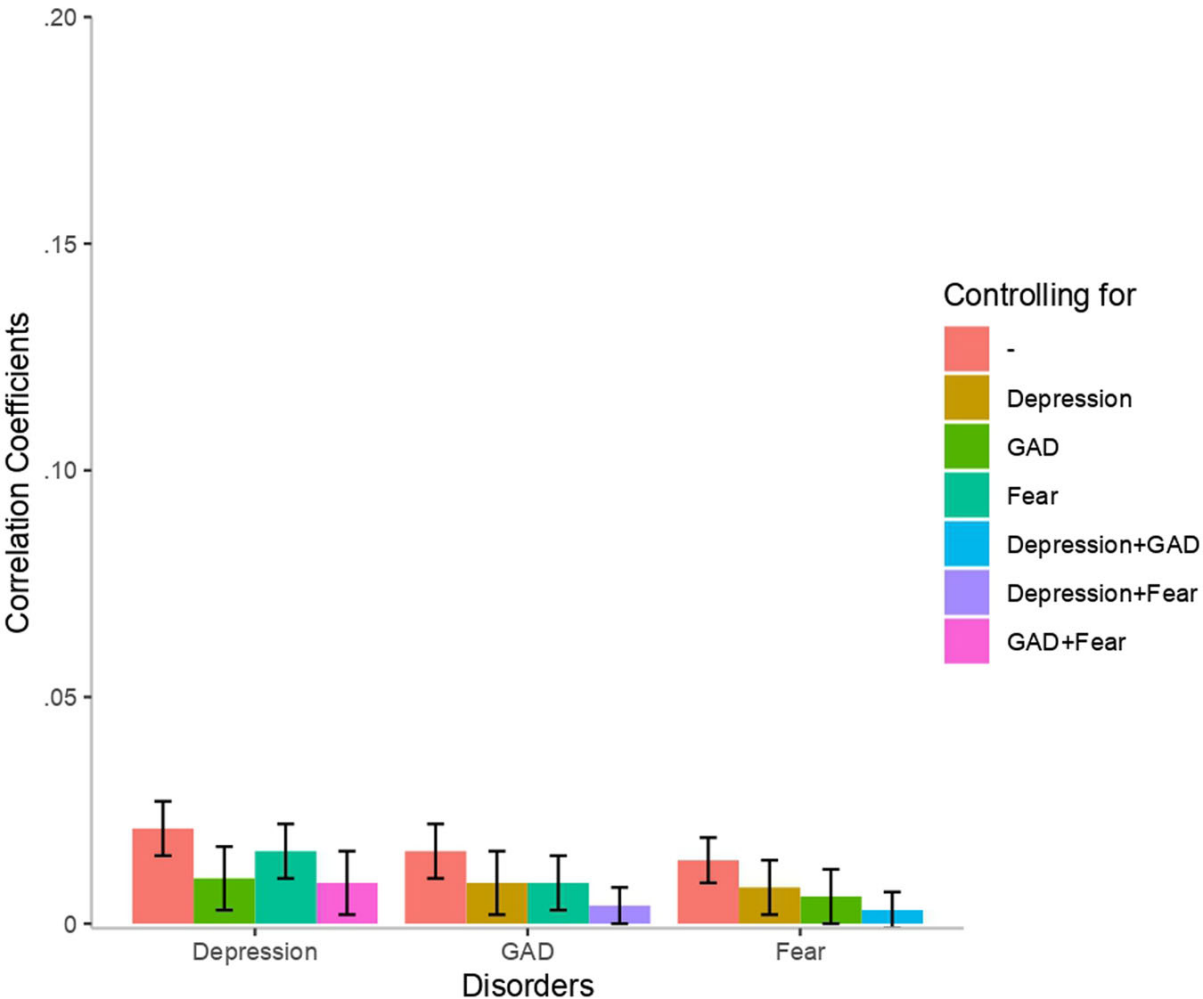
Phenotypic correlations between social isolation and disorders. 95% confidence intervals are presented as error bars. GAD, generalized anxiety disorder

Full and partial correlations between disorders and psychosocial variables are presented in Figures 1 and 2. Loneliness was moderately correlated with generalized anxiety (*r* = .25) and depression (*r* = .26), and only weakly with fear (*r* = .11). In contrast, associations with social isolation were very weak (*r* < .02) and did not allow distinguishing distress disorders from fear. Again, differences between correlations were tested with a two‐sample Z test after a Fisher r‐to‐z transformation, but none of them were statistically different.

Partial correlations showed that the association between loneliness and depression was marginally more attenuated when controlling for generalized anxiety than when controlling for fear. Similarly, the association between loneliness and generalized anxiety was more strongly attenuated when controlling for depression than when controlling for fear, suggesting that depression and generalized anxiety shared more variance with loneliness than did depression and fear. Partial correlations between loneliness and fear were weak. Partial correlations between loneliness and the unique variance of each disorder (controlling for the other two disorders) revealed stronger associations with depression and generalized anxiety than with fear. Overall, partial correlations indicate that the two distress disorders shared more variance with loneliness than either one of the distress disorder and fear. We conducted genetic correlations between disorders and psychosocial variables to document the role of SNPs in these associations (see Figures S1 and S2).

## 4 DISCUSSION

The aim of this study was to examine the role of specific genetic and psychosocial factors in validating the distress and fear classification for anxiety and depressive disorders. Using data from the UK Biobank, we provide preliminary evidence that this hierarchical structure is supported through genome‐wide genetic associations and phenotypic relationships with loneliness.

First, we found that genome‐wide genetic correlations partially supported the distress and fear model. Our results add to previous twin studies showing strong genetic correlations between depression and generalized anxiety by suggesting that this genetic association is in part accounted for by common genetic variants (Kendler et al., 1992; Roy et al., 1995). This finding provides preliminary evidence that the genetic correlation between broadly measured anxiety and depression from a recent genome‐wide study may be due to a genetic overlap between depression and generalized anxiety specifically, and not between depression and fear disorders (Anttila et al., 2018). Second, in agreement with the distinction of distress and fear disorders, we found no genetic relationship between depression and fear after controlling for general anxiety. This finding is inconsistent with twin study findings showing a genetic link between panic disorder (a fear disorder), and depression (Kendler et al., 2003). Had we tested this association between specific fear disorder and depression, we may have found similar results. However, statistical power was not sufficient to perform genetic correlations with each fear disorder separately. Finally, we found that distress and fear were not entirely distinguishable on the basis of their genetic structure since there was a genetic association between generalized anxiety and fear, independently from depression. This genetic association may be explained by a common genetic vulnerability for symptoms characterizing all anxiety disorders, for instance, excessive worry or fear about the future, which are not a core feature of depression (APA, 2013; Kessler et al., 1998). Nevertheless, at the phenotypic level, generalized anxiety and fear were only moderately associated when controlling for depression and their genetic association has to be interpreted within the scope of this moderate phenotypic association.

Next, we found that the distress and fear structure was also supported to some extent by phenotypic relationships with loneliness. Depression and generalized anxiety showed moderate associations with loneliness, while fear was only weakly correlated with loneliness. This finding provides additional evidence for a shared distress dimension and for a distinction between distress and fear disorders. It is consistent with previous studies showing that lonely individuals are more likely to have depression or generalized anxiety and adds to the current literature by indicating that loneliness may not be as strongly linked to fear disorders (Beutel et al., 2017; Hyland et al., 2018). This partial distinction with fear may be explained by the fact that loneliness is a negative feeling reflecting a subjective dissatisfaction about one's social relationships (Ernst & Cacioppo, 1999), and that negative affect could be a component more associated with distress than with fear (Gamez, Watson, & Doebbeling, 2007; Mineka et al., 1998). Moreover, it is possible that the phenotypic association between loneliness and distress disorders is driven by common genetic influences. We conducted genetic correlations between loneliness and all disorders and found that, indeed, loneliness was genetically associated with depression and generalized anxiety, respectively (see Figure S1). Future studies should examine whether SNPs associated with loneliness are shared across both distress disorders.

In contrast with loneliness, social isolation was not phenotypically correlated with any disorder. This may be explained by the fact that isolated anxious or depressed individuals are likely to refrain from seeking treatment (Mojtabai, Olfson, & Mechanic, 2002). Therefore, it is possible that isolated individuals who may have received a diagnosis had they consulted a professional have been ignored. Furthermore, some individuals do not mind living alone and prefer solitary hobbies rather than group activities (Cacioppo, Cacioppo, Capitanio, & Cole, 2015). These individuals may be satisfied with their social life and for them, social isolation may not be a risk factor for anxiety or depression. Nevertheless, our findings contribute to a debate as to whether social isolation is a risk factor for anxiety and depression. While our results are inconsistent with evidence suggesting a link between social isolation and anxiety and depression (Schwarzbach et al., 2014; Teo et al., 2013), they are coherent with other studies showing that only loneliness, and not social isolation, is a risk factor for poor mental health (Coyle & Dugan, 2012; Steptoe et al., 2013).

The strengths of this study are the large population‐based sample of the UK Biobank, the genome‐wide genetic data, and the evaluations of specific anxiety disorders and psychosocial factors. These evaluations allowed differentiating generalized anxiety from the other anxiety disorders and loneliness from social isolation. However, our study bears some limits. First, we were unable to examine fear disorders separately to have sufficient statistical power to perform genomic analyses. Second, evaluations of depression and generalized anxiety were likely to be more accurate than the single‐item measure for fear disorders. However, measures were based on available data in the UK Biobank and allowed us to have specific measures of all anxiety disorders. Third, this study was observational and did not allow inference on the direction of association between psychosocial factors and the disorders. It is possible that loneliness is a consequence of the disorders rather than a risk factor or that a gene‐environment correlation is at play (see the Supplementary material). Future research should examine this question within a longitudinal perspective. Fourth, it is possible that we found strong genetic correlations because both genetic and phenotypic data came from the same sample. Future research should aim at replicating these findings across different samples. Finally, participants of the UK Biobank are on average healthier and more likely to live in less socioeconomically deprived areas than the general UK population (Fry et al., 2017) and only individuals from European ancestry were included in genetic analyses. This limits the generalization of results to the general population and to individuals of other ancestry.

## 5 CONCLUSION

This study provides preliminary evidence supporting the distress and fear classification of anxiety and depression. Above and beyond symptom and diagnostic characteristics, findings revealed that measured genetic variants and loneliness may accurately distinguish distress from fear. There is still a need to further clarify the etiological architecture of the distress and fear model, but our study contributes to the current state of knowledge on potential mechanisms underlying this structure.

## CONFLICT OF INTERESTS

The authors declare that there are no conflict of interests.

## Supporting information

Supporting information

## Data Availability

The data (GWAS summary statistics) that support the findings of this study are openly available at https://phenviz.navigome.com/downloads.
