# Supplementary material for: The genetic and environmental hierarchical structure of anxiety and depression in the UK Biobank
Source: Depress Anxiety. 2020 Jan 17;37(6):512–20. doi: 10.1002/da.22991 (PMC7318128; doi:10.1002/da.22991)
Supplement: Supplementary file 1 — Supporting information [file DA-37-512-s001.docx]

**Supplementary material**

[**Supplementary figures** 2](#_Toc14441790)

[*Figure S1. Genetic correlations between loneliness and disorders* 3](#_Toc14441791)

[*Figure S2. Genetic correlations between social isolation and disorders* 4](#_Toc14441792)

[**Supplementary tables** 5](#_Toc14441793)

[*Table S1. Phenotypic correlations between disorders, loneliness, and social isolation* 5](#_Toc14441794)

[*Table S2. Genetic correlations between disorders, loneliness, and social isolation* 6](#_Toc14441795)

[**Additional information on measures for loneliness and social isolation** 7](#_Toc14441796)

[*Table S3. Coding of socially isolated participants* 8](#_Toc14441797)

# **Supplementary figures**

We conducted full and partial genetic correlations between disorder phenotypes and both loneliness and social isolation (see Figures S1 and S2. These genetic correlations were based on GWAS summary statistics for all phenotypes. Summary statistics for loneliness and social isolation are available at (<https://phenviz.navigome.com/downloads>).

All genetic correlations with loneliness, except for fear controlling for depression and generalized anxiety, were statistically significant (see Figure S1). Genetic associations between loneliness and psychiatric disorders were similar to phenotypic correlations. Loneliness and distress disorders had stronger genetic associations than loneliness and fear. The genetic associations between distress disorders and loneliness remained strong after controlling for fear, but they were more strongly attenuated after controlling for the other distress disorder. This suggests that loneliness had a larger genetic overlap with distress disorders than with fear. Finally, no significant genetic correlation was found for social isolation (see Figure S2).

**
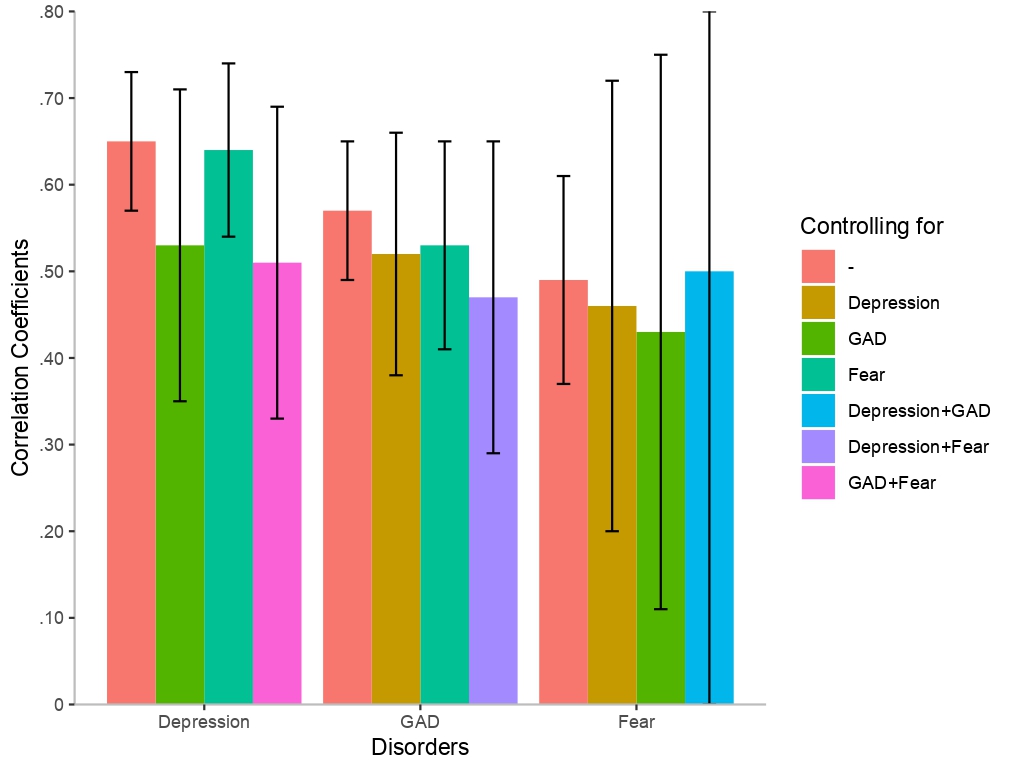
**

| *Figure S1. Genetic correlations between loneliness and disorders* |
| --- |
| Confidence intervals (95%) are presented as error bars. |


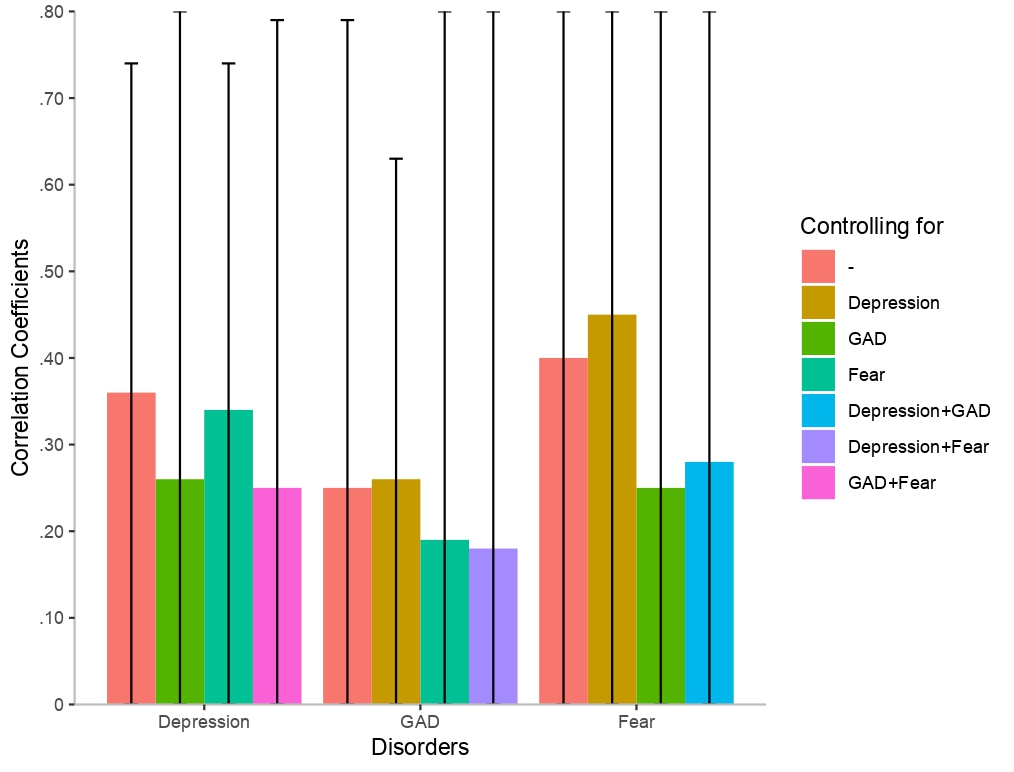


| *Figure S2. Genetic correlations between social isolation and disorders* |
| --- |
| Confidence intervals (95%) are presented as error bars. |

# **Supplementary tables**

| *Table S1. Phenotypic correlations between disorders, loneliness, and social isolation* | | |
| --- | --- | --- |
|  | Loneliness (95% C.I.) | Social isolation (95% C.I.) |
| Depression | .26 (.26–.27) | .02 (.01–.03) |
| Depression – (GAD) | .16 (.15–.17) | .01 (.00–.02) |
| Depression – (Fear) | .23 (.22–.24) | .02 (.01–.03) |
| Depression – (GAD+Fear) | .16 (.15–.17) | .01 (.00–.02) |
| GAD | .25 (.24–.26) | .02 (.01–.03) |
| GAD – (Depression) | .14 (.13–.15) | .01 (.00–.02) |
| GAD – (Fear) | .20 (.19–.21) | .01 (.00–.02) |
| GAD – (Depression+Fear) | .11 (.10–.12) | .00 (.00–.00) |
| Fear | .11 (.11–.12) | .01 (.01–.02) |
| Fear – (Depression) | .06 (.05–.07) | .01 (.00–.02) |
| Fear – (GAD) | .05 (.04–.06) | .01 (.00–.02) |
| Fear – (Depression+GAD) | .04 (.03–.05) | .00 (.00–.00) |

| *Table S2. Genetic correlations between disorders, loneliness, and social isolation* | | |
| --- | --- | --- |
|  | Loneliness (95% C.I.) | Social isolation (95% C.I.) |
| Depression | .65 (.57–.73) | .36 (-.02–.74) |
| Depression – (GAD) | .53 (.35–.71) | .26 (-.28–.80) |
| Depression – (Fear) | .64 (.54–.74) | .34 (-.06–.74) |
| Depression – (GAD+Fear) | .51 (.33–.69) | .25 (-.29–.79) |
| GAD | .57 (.49–.65) | .25 (-.29–.79) |
| GAD – (Depression) | .52 (.38–.66) | .26 (-.11–.63) |
| GAD – (Fear) | .53 (.41–.65) | .19 (-.45–.83) |
| GAD – (Depression+Fear) | .47 (.29–.65) | .18 (-.70–1.00) |
| Fear | .49 (.37–.61) | .40 (-.02–.82) |
| Fear – (Depression) | .46 (.20–.72) | .45 (-.19–1.00) |
| Fear – (GAD) | .43 (.11–.75) | .25 (-.51–1.00 |
| Fear – (Depression+GAD) | .50 (-.20–1.00) | .28 (-.28–.84) |

# **Additional information on measures for loneliness and social isolation**

Loneliness and social isolation were evaluated from self-reports in the first wave of the UK Biobank (i.e., not the mental health questionnaire). The loneliness measure was based on the following item: *Do you often feel lonely?* (*Yes*, *no*, *do not know*, *prefer not to answer*).

Social isolation was a combination of three questions. The first question was: *Including yourself, how many people are living together in your household?* Participants answered this question by giving a number. Next, the frequency of social interactions was evaluated with two questions. One of which was: *Which of the following do you attend once a week or more often?* Participants answered at least one among the following choices: *Sports club or gym*, *pub or social club*, *religious group*, a*dult education class*, o*ther group activity*, *none of the above*, *prefer not to answer*. The last question was: *How often do you visit friends or family or have them visit you?* Participants answered one of the following choices: *Almost daily*, *two to four times a week*, *about once a week*, *about once a month*, *once every few months*, *never or almost never*, *no friends/family outside household*, *do not know*, *prefer not to answer*). Participants were considered socially isolated if they were living alone and did not have frequent social interactions. Possible answers for classifying participants as socially isolated are presented in Table S3. When participants lived with at least one other person, they were not considered socially isolated. They were also not considered socially isolated if they lived alone, but either had frequent weekly social activities or had visits with friends and family at least once a month.

| *Table S3. Coding of socially isolated participants* | | |
| --- | --- | --- |
| “Including yourself, how many people are living together in your household?” | “Which of the following do you attend once a week or more often?” | “How often do you visit friends or family or have them visit you?” |
| 1 | *None of the above* | *Once every few months* |
| 1 | *None of the above* | *Never or almost never* |
| 1 | *None of the above* | *No friends/family outside household* |
| 1 | *None of the above* | *Do not know* |
| 1 | *None of the above* | *Prefer not to answer* |
| 1 | *None of the above* | Missing |
| 1 | *Prefer not to answer* | *Once every few months* |
| 1 | *Prefer not to answer* | *Never or almost never* |
| 1 | *Prefer not to answer* | *No friends/family outside household* |
| 1 | *Prefer not to answer* | *Do not know* |
| 1 | *Prefer not to answer* | *Prefer not to answer* |
| 1 | *Prefer not to answer* | Missing |
| 1 | Missing | *Once every few months* |
| 1 | Missing | *Never or almost never* |
| 1 | Missing | *No friends/family outside household* |
| 1 | Missing | *Do not know* |
| 1 | Missing | *Prefer not to answer* |
| 1 | Missing | Missing |
